# Vesicle Size Regulates Nanotube Formation in the Cell

**DOI:** 10.1038/srep24002

**Published:** 2016-04-07

**Authors:** Qian Peter Su, Wanqing Du, Qinghua Ji, Boxin Xue, Dong Jiang, Yueyao Zhu, He Ren, Chuanmao Zhang, Jizhong Lou, Li Yu, Yujie Sun

**Affiliations:** 1 State Key Laboratory of Membrane Biology, Biodynamic Optical Imaging Center, School of Life Sciences, Peking University, Beijing 100871, China; 2 State Key Laboratory of Membrane Biology, Tsinghua-Peking University Joint Center for Life Sciences, School of Life Sciences, Tsinghua University, Beijing 100084, China; 3 Laboratory of RNA Biology, Institute of Biophysics, Chinese Academy of Sciences, Beijing 100101, China; 4 University of Chinese Academy of Sciences, Beijing 100049, China; 5 School of Life Sciences, Peking University, Beijing 100871, China

## Abstract

Intracellular membrane nanotube formation and its dynamics play important roles for cargo transportation and organelle biogenesis. Regarding the regulation mechanisms, while much attention has been paid on the lipid composition and its associated protein molecules, effects of the vesicle size has not been studied in the cell. Giant unilamellar vesicles (GUVs) are often used for *in vitro* membrane deformation studies, but they are much larger than most intracellular vesicles and the *in vitro* studies also lack physiological relevance. Here, we use lysosomes and autolysosomes, whose sizes range between 100 nm and 1 μm, as model systems to study the size effects on nanotube formation both *in vivo* and *in vitro*. Single molecule observations indicate that driven by kinesin motors, small vesicles (100–200 nm) are mainly transported along the tracks while a remarkable portion of large vesicles (500–1000 nm) form nanotubes. This size effect is further confirmed by *in vitro* reconstitution assays on liposomes and purified lysosomes and autolysosomes. We also apply Atomic Force Microscopy (AFM) to measure the initiation force for nanotube formation. These results suggest that the size-dependence may be one of the mechanisms for cells to regulate cellular processes involving membrane-deformation, such as the timing of tubulation-mediated vesicle recycling.

Biological membrane nanotubes are tubular structures that are commonly observed within the cell and between cells. The formation and dynamics of nanotubes are increasingly recognized to play important roles in a multitude of biological progresses, such as endosomal antigen delivery in polarized T-cells^1^, transportation between ER and Golgi^2^ and between Golgi and the plasma membrane^3^. We recently also demonstrated the role of nanotube dynamics in autophagic lysosome reformation during autophagy^4^,^5^ and mitochondrial network remodeling^6^. Nanotube formation is usually an active process that requires works. McMahon and Gallop have reviewed the factors that drive biological membrane deformation, including the lipid composition, membrane proteins and scaffolding proteins as well as cytoskeleton and its associated motor proteins^7^. Up to now, mechanistic understanding of membrane tubulation is mainly based on *in vitro* reconstitution assays, which comprise two approaches^8^. In the first approach, components that are thought to be involved in membrane tubulation are either synthesized, purified or even kept in cell extracts and used to reconstitute the tubulation process. This approach has helped to make reduction of required components for nanotube formation^9^. As an example, such assays have established that kinesin motors and microtubules are sufficient to induce membrane tubes from vesicles in the absence of any other machinery or proteins^10^. The other approach focuses more on the mechanical response of membrane tubulation and is thus often conducted with model liposomes, namely Giant Unilamellar Vesicles (GUVs)^11^,^12^. Usually, direct single vesicle manipulation techniques are used in these assays, including hydrodynamic flow^13^, micropipettes^14^ and optical tweezers^15^,^16^. As these techniques allow to control the load precisely, the mechanical response of membrane tubulation may be studied in details.

Despite all the findings, it is not clear at what extent the size of a vesicle affects its tubulation. This question is of great interest as the size of vesicles and organelles is heterogeneous in the cell. Although a previous *in vitro* study indicates that the force required to initiate tubulation is scaled with the vesicle size, the conclusion was limited to GUVs which are about 20 μm in diameter^15^, much larger than most intracellular vesicles and organelles. In contrast, little has been accomplished for understanding tubulation of small vesicles that are more physiologically relevant.

In this work, our goal is to determine how vesicle size affects membrane tubulation within living cells using lysosomes and autolysosomes as a set of model systems. Autolysosomes are degradative compartments formed by fusion of an autophagosome and multiple lysosomes during autophagy. We recently discovered that at the late stage of autophagy, tubular structures are extruded and pinched off from autolysosomes to form small proto-lysosomes, which become functional lysosomes after a maturation process. This process, namely autophagic lysosomal reformation (ALR), is crucial for cells to retain their lysosome level when they exit the autophagic stage^4^. While the membrane composition of autolysosomes is similar with lysosomes, their sizes are quite different. It is known that the range of lysosome diameter is between 50 nm and 500 nm^17^. For autolysosomes, their size ranges from a few hundred nanometers to several micrometers, larger and more varied than that of lysosomes^17^. We speculate that cells may use vesicle size to distinguish lysosomes and autolysosomes in promoting tubule formation specifically on autolysosomes. To prove this idea, we first quantify the tubulation percentage of lysosomes and autolysosomes in the cell. The *in vivo* observations reveal that autolysosomes show much higher probability of tubulation than lysosomes. The size-dependence hypothesis is further enforced by a sucrose-induced enlargement method in the cell. In addition, with the knowledge that tubulation of both autolysosomes and lysosomes is driven by associated kinesin motors, we reconstitute the tubulation process *in vitro* using purified lysosomes, autolysosomes and artificial liposomes. Precise tuning of the kinesin motor concentration allows to separate the size effect on tubulation from other factors. Lastly, we apply Atomic Force Microscopy (AFM) to quantitatively measure the force barrier during the tubulation process and carry a simple computation based on the measurements.

Overall, these results suggest that vesicle size indeed affects the tubulation probability for lysosomes and autolysosomes, whose size ranges between 50 nm and several micrometers. Our assays built for lysosomes and autolysosomes may be easily extended to other vesicle deformation systems. Importantly, the size-dependence effect may be one of the mechanisms for the cell to regulate cellular processes involving membrane-deformation.

## Results

### *In vivo* tubulation of lysosomes and autolysosomes is size-dependent

**Figure 1:**
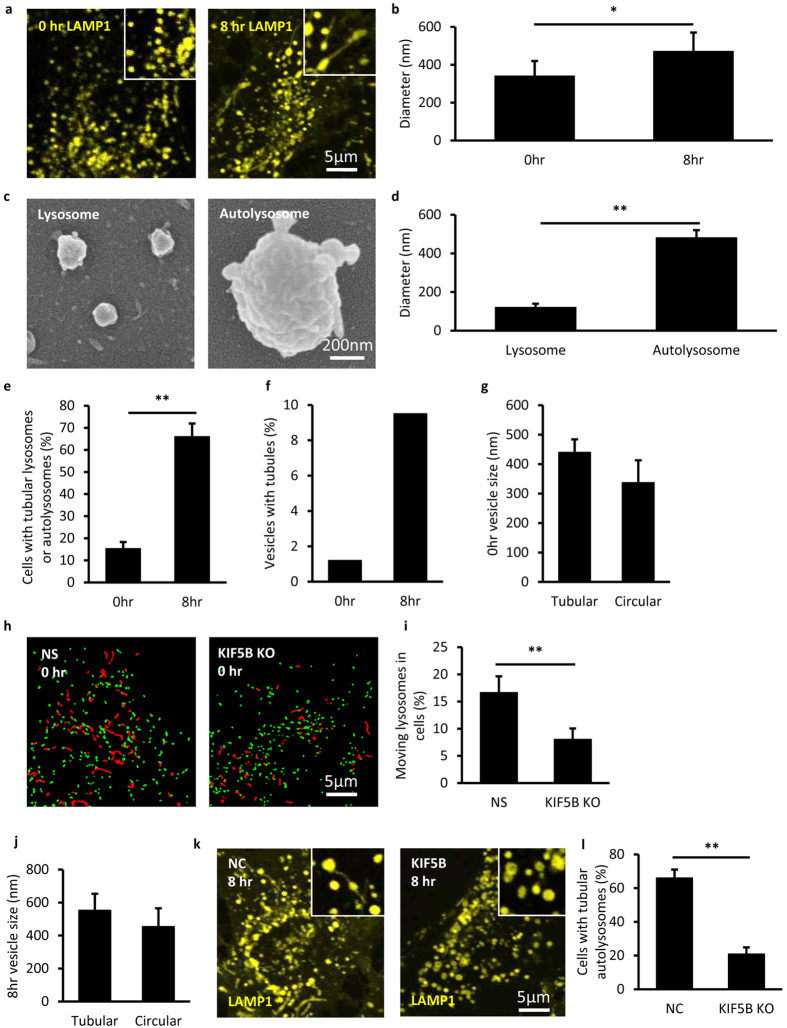
Size dependent lysosome motion and autolysosome tubulation driven by KIF5B *in vivo*. **(a)** NRK cells stably expressing LAMP1-mCherry (yellow) were starved for 0 hr and 8 hr. At 0 hr all the LAMP-1 positive vesicles are relative small and circular, while at 8 hr some large and tubular vesicles appear. Scale bar, 5 μm. (**b**) The diameter of the vesicles at 0 hr and 8 hr was analyzed from the fluorescent images in (**a**). n > 300. Error bars indicate the s.d. This value, measured with optical fluorescence microscopy, is close to the resolution limited by optical diffraction and may not reflect the real sizes^18^. **(c)** Lysosomes and autolysosomes were purified from NRK cells and the quality of purified vesicles was monitored by SEM. Scale bar, 200 nm. **(d)** The diameter of the vesicles was analyzed from the SEM images in (**c**). n > 300. Error bars indicate the s.d., p < 0.01. **(e)** Cells from (**a**) were assessed for tubular lysosomes or autolysosomes and quantified. n = 100 cells from three independent experiments. Error bars indicate the s.d., p < 0.01. **(f)** Percentage of vesicles with tubular structure from (**a**) were assessed. n > 5000. **(g)** The diameter of the vesicles from 0 hr cells in (**a**) was analyzed. n > 10000 lysosomes. Error bars indicate the s.d. **(h)** Lysosomes in NRK cells were labeled with LysoTracker and tracked on a Spinning Disk Confocal Microscope. The lysosomes whose moving distance is longer than 1 μm (red trajectories) and the lysosomes whose moving range less than 1 μm (green dots) were tracked in normal NRK cells and KIF5B KO cells. Scale bar, 5 μm. **(i)** The percentage of moving lysosomes in cells from (**k**) were determined. n > 5000 lysosomes from three independent experiments. Error bars indicate the s.d., p < 0.01. **(j)** The diameter of the vesicles from 8 hr cells in (**a**) was analyzed. n > 5000 autolysosomes. Error bars indicate the s.d. **(k)** NRK cells stably expressing LAMP1-mCherry were transfected with nonspecific (NS)-RNAi or KIF5B-RNAi and starved for 8 hr. Scale bar, 5 μm. **(l)** Cells from (**i**) were assessed for tubular autolysosomes after starvation and quantified. n = 100 cells from three independent experiments. Error bars indicate the s.d., p < 0.01.

We first used lysosomes and autolysosomes as model systems to study the size-dependence of vesicle tubulation in the cell. It is known that after starvation for 8 hr, lysosomes in NRK cells are mostly taken up by autolysosomes^4^. Therefore, NRK cells stably expressing LAMP1-mCherry starved for 0 hr and 8 hr were used for quantifications of lysosomes and autolysosomes, respectively. Fluorescence microscopy (FM) images show that lysosomes (diameter 340 ± 80 nm, s.d., N = 12870) were smaller than autolysosomes (diameter 470 ± 100 nm, s.d., N = 5599), see Fig. 1a,b. As optical diffraction limits the spatial resolution of fluorescence imaging at about 300 nm^18^, the values obtained with FM may not reflect the real vesicle size. We then used scanning electron microscopy (SEM) to measure the size of purified lysosomes and autolysosomes (Fig. 1c,d). The data show that the mean size of autolysosomes is similar with the measurements in the cell, whereas the size of lysosomes was only about 100 nm, much smaller than that measured in the cell using FM. Therefore, the actual size of lysosomes should be around 100 nm as measured by SEM. In addition, the purity of the lysosomes and autolysosomes was quantified by western blot towards LAMP2, LC3, GM130 and Calnexin (Fig. S1a).

We next quantified the tubulation events as a function of the sizes of lysosomes and autolysosomes. For NRK cells under 0 hr starvation, lysosomes are the majority and only 15.31% cells were found to contain tubular lysosomes (Fig. 1e left column) and less than 2% of all vesicles formed tubules (Fig. 1f left column). Notably, the average diameter of these tubulated lysosomes was found to be similar with the mean size of autolysosomes and remarkably larger than that of un-deformed lysosomes (Fig. 1g). For the un-deformed lysosomes, time lapse imaging revealed that they mainly took directional, long range movement, which could be significantly reduced by KIF5B knockdown (Fig. S1b, Fig. 1h,i). In clear contrast, LAMP1 positive autolysosomes in NRK cells starved for 8 hours exhibited much higher percentage of tubulation processes (Fig. 1e right column & Fig. 1f right column). Interestingly, the average diameter of tubulated autolysosomes was found to be about 20% larger than the mean size of all autolysosomes (Fig. 1j). Knockdown experiments also proved that KIF5B is responsible for autolysosome tubulation (Fig. 1k,l). The level of KIF5B mRNA and protein were confirmed by qPCR and western blot, respectively (Fig. S1c,d).

To further clarify this size-dependence effect, we used the sucrose-swelling method^19^ to increase the size of lysosomes and autolysosomes without altering their compositions or modifications. The data show that as sucrose-treatment increased the vesicle size, the fraction of tabulated vesicles was also increased accordingly (Fig. S2a–c). Note that the quantitative western blotting (Fig. S2d) and single layer lipid surface measurements (Fig. S2e) excluded the possibility that the increased tubulation fraction is due to the alteration of kinesin motor density on the membrane surface.

Taken together, these observations and analysis suggest that tubulation probability of lysosomes and autolysosomes in the cells is dependent on their sizes.

### The size-dependence of vesicle tubulation is confirmed by *in vitro* reconstitution assays

In order to separate the size-dependence effect from the complication from other factors in the cell, such as the types and numbers of vesicle-associated motors, we set up *in vitro* assays to reconstitute the tubulation process using purified lysosomes, autolysosomes as well as synthesized liposomes. The *in vitro* reconstitution system was mainly composed with recombinant KIF5B motor proteins, surface-bound microtubule tracks and vesicles, see Materials and Methods for details.

We first used purified lysosomes, autolysosomes (Fig. 1c) and full-length KIF5B (Fig. S3a) to further confirm the results obtained in the cellular environment. The purified lysosomes and autolysosomes were found to be still capable of recruiting full-length KIF5B motors *in vitro* (Fig. S3b).

**Figure 2:**
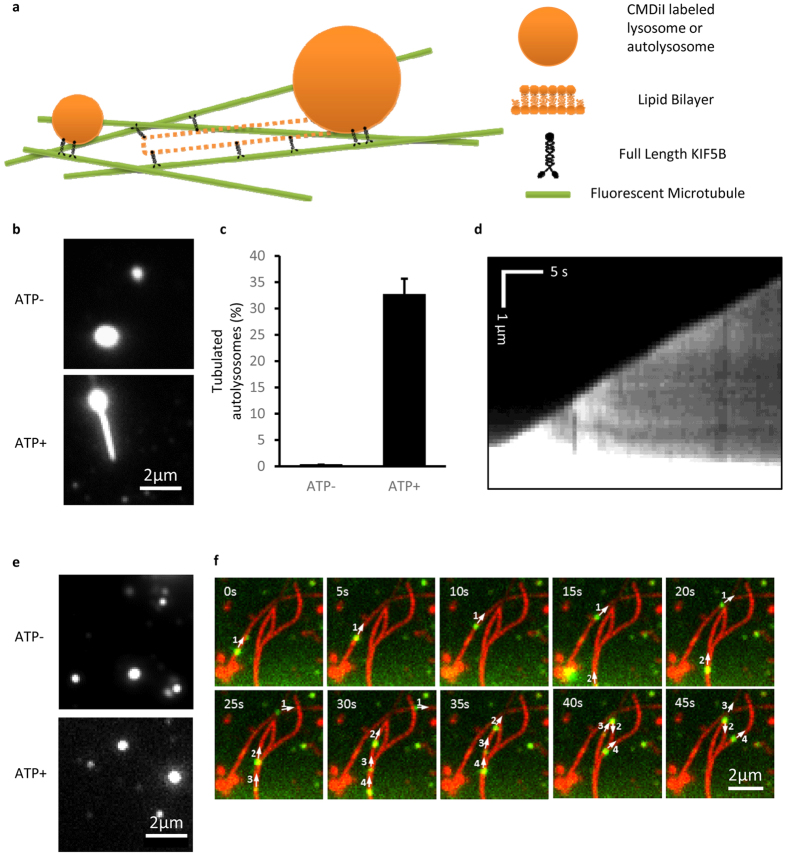
Lysosome motion and autolysosome tubulation driven by full length KIF5B using *in vitro* reconstitution assays. **(a)** Schematic diagram of *in vitro* autolysosome tubulation. Purified lysosomes or autolysosomes were stained with CMDiI and incubated with full-length KIF5B, transferred into flow chamber channels coated with microtubules and visualized in the presence of ATP. **(b)** Purified autolysosomes were stained by CM-DiI, incubated with full-length KIF5B, and transferred into flow chamber channels coated with microtubules. The images were collected with a Nikon TIRF microscope. Scale bar, 2 μm. **(c)** The percentage of tubulated autolysosomes from (**b**) was determined. n > 100 from three independent experiments. Error bars indicate the s.d. **(d)** Kymograph of the tubulation process, scale bar, horizontal 5 s and vertical 1 μm. **(e)** Purified lysosomes were labeled by CM-DiI, incubated with KIF5B, and transferred into flow chamber channels coated with microtubules. The images were collected with a Nikon TIRF microscope. Scale bar, 2 μm. **(f)** Time-lapse sequences of *in vitro* lysosome motion in the presence of ATP. Scale bar, 2 μm.

Thus, the reconstituted tubulation was set up by incubating full-length KIF5B and fluorescently labeled lysosomes or autolysosomes in the flow chambers that contained surface-bound microtubule tracks (Fig. 2a). In the presence of ATP, kinesin motors were found to be able to pull tubules out of the purified autolysosomes (Fig. 2b). Figure 2c shows that more than 30% of the autolysosomes formed tubules (n > 100). The kymograph of one autolysosome tubulation event exhibits a constant speed of about 140 nm/s. In contrast, purified lysosomes were hardly found to form tubules in spite of the presence of kinesin motors and ATP (Fig. 2e). Instead, most lysosomes were moving along microtubule tracks with an average speed of 506 nm/s (Fig. 2f). These *in vitro* results recapitulate the size-dependent tubulation observed in the cell (Fig. 1a,h).

**Figure 3:**
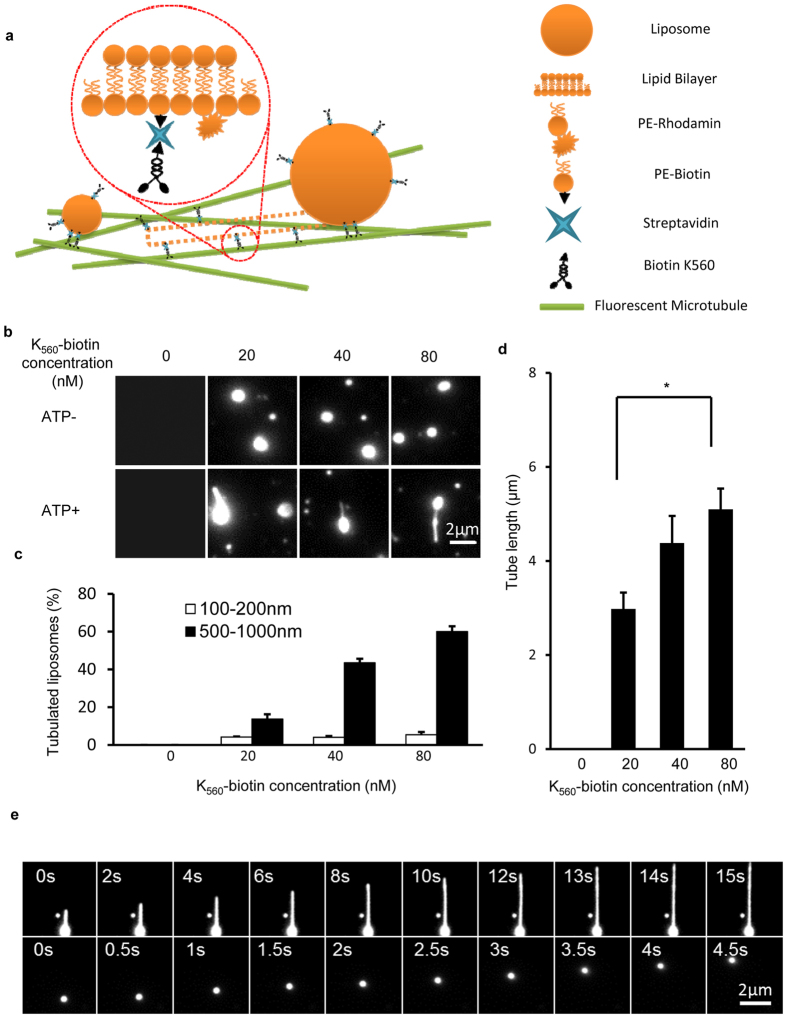
Size dependent liposome motion and tubulation driven by truncated KIF5B *in vitro*. **(a)** Schematic diagram of the *in vitro* liposome tubulation assay. The brain polar lipid mixture was supplemented with 20% PE-biotin and 1% PE-rhodamine B, and the solution was extruded through a 100 nm or 1000 μm pore polycarbonate filter to generate different size liposomes. K_560_-biotin was mixed with streptavidin and liposomes, and the resulting complexes were transferred into flow chamber channels pre-incubated with microtubules. Liposome tubulation was visualized in the presence of ATP. **(b)** The effect of K_560_-biotin concentration on *in vitro* liposome tubulation. Different concentrations of K_560_-biotin (0, 20, 40 and 80 nM) were prepared, mixed with streptavidin (1:1 molar ratio) and then incubated with liposome solution on ice. The liposome concentration was held fixed at 28 μg/ml. The resulting mixtures were transferred to flow chamber channels coated with microtubules, and images were collected with a Nikon TIRF microscope. Scale bar, 2 μm. **(c)** The percentage of tubulated liposomes in (**b**) was determined. n > 100 from three independent experiments. Error bars indicate the s.d. **(d)** Tube length from (**b**) was assessed and quantified. n > 100 from three independent experiments. Error bars indicate the s.d. **(e)** Time-lapse sequence of liposome (500–1000 nm) tubulation and liposome (100–200 nm) motion were collected in the presence of ATP. Scale bar, 2 μm.

Considering other potential differences between lysosomes and autolysosomes besides their sizes, we further sought to reconstitute the size-dependent tubulation using synthesized liposomes sized 100–200 nm and 500–1000 nm, which are the typical diameters of lysosomes and autolysosomes, respectively. A biotinylated KIF5B truncation (K_560_-biotin) was prepared (Fig. S4a) and allowed to bind liposomes through the biotin-streptavidin interaction (Fig. 3a and Fig. S4b).

The tubulation probability was quantified as a function of kinesin concentration and liposome diameters. Figure 3b indicates that in the presence of ATP, 500–1000 nm sized liposomes were able to form tubules with 20–80 nM kinesin motors. Importantly, although the tubulation percentage of the 500–1000 nm liposomes increased with the kinesin concentration, the 100–200 nm liposomes rarely formed tubules and no apparent dependence on the kinesin concentration was found either (Fig. 3c). For the 500–1000 nm liposomes that formed tubules, we also found the tubule length became longer as the concentration of kinesin increased (Fig. 3d). As a typical scenario of the dynamic liposome tubulation process, Fig. 3e demonstrates that driven by the kinesin motor proteins, a small vesicle (100–200 nm) was transported along the microtubule tracks at a speed of about 500 nm/s, while the large vesicle (500–1000 nm) was deformed to form tubules at a growth rate of about 100 nm/s. These quantitative studies of liposomes support that the differences in the tubulation probability between lysosomes and autolysosomes are mainly caused by their differed sizes.

### Quantification of the tubulation force using Atomic Force Microscopy (AFM)

**Figure 4:**
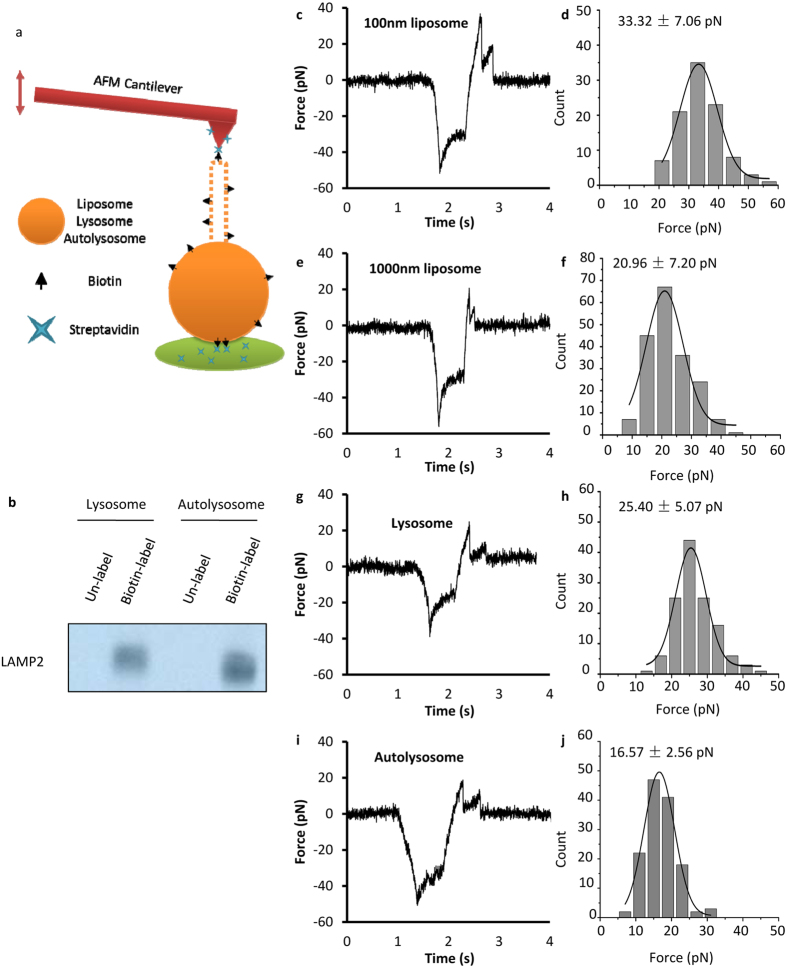
AFM force spectroscopy test on liposomes, lysosomes and autolysosomes. **(a)** Schematic diagram of AFM spectroscopy test on biotin labeled vesicles. The brain polar lipid mixture was supplemented with 20% PE-biotin and 1% PE-rhodamine B, and the solution was extruded through 100 nm and 1000 nm pore polycarbonate filter to generate different size liposomes. The lysosomes and autolysosomes were labeled with NHS conjugated Biotin. **(b)** Labeled lysosomes and autolysosomes were crosslinked with streptavidin beads and detected by western blot towards LAMP2. **(c,d)** Force (pN) vs time (s) curve and histogram of the first peak (pN) for 100 nm liposomes. **(e,f)** Force (pN) vs time (s) curve and histogram of the first peak (pN) for 1000 nm liposomes. **(g,h)** Force (pN) vs time (s) curve and histogram of the first peak (pN) for lysosomes. **(i,j)** Force (pN) vs time (s) curve and histogram of the first peak (pN) for autolysosomes.

We next sought to characterize the force required for vesicle tubulation in order to obtain more quantitative understanding of the size-dependent effect. AFM is a widely used technique for single molecule force studies in the range of several to a few hundred pN. We used a home-made AFM^20^ to measure the force required to generate tubules from liposomes, lysosomes and autolysosomes. The biotin-streptavidin interaction was used to tether the AFM tip to the surface-bound vesicles and the tip was ramped repeatedly at a speed of 200 nm/s to pull tubules out of the vesicle (Fig. 4a,b). Like the micro-pipette measurements on GUVs^15^,^16^, the first peak in the force-time curve is the force barrier to initiate the tubule (Fig. 4c,e,g,i). It was found that the peak dropped sharply as soon as the tubule was initiated, and then the force gradually increased as the AFM tip kept pulling until the tubule broke. We quantified the barrier force to initiate the tubule for liposomes (diameter of 100 nm (Fig. 4c,d) and 1000 nm (Fig. 4e,f)), lysosomes (Fig. 4g,h) and autolysosomes (Fig. 4i,j). It shows that the 100 nm liposomes required about 33 pN for the initiation while the 1000 nm liposomes only needed 21 pN force. For the purified lysosomes and autolysosomes, it took about 17 pN force for autolysosomes, which is about 30% less than that required for lysosomes. These force measurements are consistent with the tubulation probability revealed in Figs 2 and 3.

## Discussion

An important mark of eukaryotic cells is the emergence of membrane-bound cellular structures, including organelles and vesicles. Their dynamics, such as fusion, fission and transportation, are essential for organelle biogenesis and cell homeostasis. Among the different parameters of membrane structures, vesicle sizes play important roles in various cellular processes. For instance, Bandyopadhyay *et al*. have reported that lysosome transport dynamics is dependent on the lysosome diameter^21^.

Compared with previous *in vitro* studies on GUVs, our work provides the first set of direct evidence to support the physiological relevance of vesicle sizes in regulation of nanotube formation and its dynamics. Firstly, instead of GUVs which are much larger than most intracellular vesicles and organelles, studies of small vesicles such as lysosomes and autolysosomes *in vivo* are more relevant to physiological conditions. Secondly, *in vitro* reconstitution using purified lysosomes, autolysosomes and artificial liposomes separates the size effect from other factors, providing solid evidence for size-dependent tubulation of small vesicles. Thirdly, the size-dependence of tubulation provides an interesting viewpoint to understand how kinesin motors choose to pull tubules out of autolysosomes but not lysosomes even though lysosomes also have associated kinesin motors. This is important because cells need certain mechanisms to control the regeneration of lysosomes specifically from autolysosomes at the right time during autophagy. In this case, an autolysosome increases in size as multiple lysosomes are fused with an autophagosome in the early stage of autophagy. In the late stage of autophagy, autolysosomes become so large that they start to form nanotubes and go through ALR for lysosome recycling, which is accompanied with decrease in the vesicle size. Note that this size-dependent timing of ALR does not exclude the effects of other biochemical changes on the autolysosome. Arguably, in addition to the size-dependent effect on herein studied tubulation of lysosomes and autolysosomes, this physical principle-based regulation mechanism may be widely used by the cell on other cellular processes, for instance the fragmentation and reformation of organelles with network morphology, such as endosomal antigen delivery in polarized T-cells^1^, transportation between ER and Golgi^2^ and between Golgi and the plasma membrane^3^, mitochondria network remodeling during cell cycle^6^, and lysosome transport dynamics^21^. Lastly, this work provides useful data for theoretical modeling of small vesicle deformation. As a matter of fact, although the analytical solutions for large membrane structures such as red blood cells and GUVs have been established, little has been done for sub-micron vesicles^8^. The main challenge is that several important approximations in describing tubulation of large vesicles no longer hold effective for small vesicles (Supplementary Materials). Nonetheless, our sketchy derivation suggested that as the vesicle size decreases, the tethering force does increase due to the increased excess surface area (Supplementary Materials). Further efforts are apparently required to obtain analytical expressions or numerical solutions for small vesicles. Besides increased excess surface area, the size dependence of vesicle tubulation may also be resulted from other effects, including motor density, number of engaged motors and counter force, see Supplementary Materials for further discussion.

In summary, we utilized lysosomes and autolysosomes as model systems to study how vesicle sizes affect membrane nanotube formation. The *in vivo* observations of lysosomes and autolysosomes revealed that autolysosomes have a much higher tubulation rate than lysosomes. The *in vitro* reconstitution eliminated other possible factors that may affect the tubulation rate in the cell and suggested the size-dependence of vesicle tubulation. Further, we confirmed the hypothesis using synthesized liposomes with sizes that are similar with lysosomes and autolysosomes. Additionally, AFM pulling measurements provided a quantitative characterization of the force barrier for pulling tubules out of these small vesicles. Overall, this systematical study provides strong evidence that the probability of tubulation is dependent on the size of the vesicles in the cell.

## Materials and Methods

### Reagents and Materials

All lipids (brain polar lipids (141101), PE-biotin (870285 P) and PE-rhodamineB (810158 P)) were purchased from Avanti Polar Lipids. Streptavidin-conjugated beads (M280), LysoTracker®Red DND-99 (L-7528) and CM-DiI (C-7000) were obtained from Molecular Probes - Life Technologies. HTS tubulin (HTS03-A), GTP (BST06-001) and Taxol (TXD01) were obtained from Cytoskeleton. Anti-LAMP1 (L1418) antibody and Anti-LAMP2 (L0668) antibody were purchased from Sigma-Aldrich. Anti-GFP (11814460001) antibody and anti-His6 (11922416001) antibody were from Roche. Anti-GST (ab19256) antibody and anti-tubulin antibody were from Abcam. The BirA enzyme was a gift from Dr. Xiangdong Li, Institute of Zoology, CAS. Streptavidin (85878) and other chemicals were purchased from Sigma Aldrich.

### Cell Culture and Transfection

NRK cells were obtained from the American Type Culture Collection (ATCC) and cultured in DMEM (Life Technologies) medium supplemented with 10% FBS (Gibco) in 37 °C incubator and 5% CO_2_. Cells were transfected with 200 pmol RNAi or a total of 2 μg DNA through Amaxa nucleofection using solution T and program X-001. Then cells were cultured in growth medium for further analysis.

### Sucrose Swelling Assay

NRK cells stably expressing CFP-LC3 and LAMP1-mCherry Red were cultured in full medium containing DMEM and 10% fetal bovine serum (FBS). To enlarge mature lysosomes, cells were subsequently transferred to the medium with 0.05 M sucrose and grown for 20 hr. To observe the effect of sucrose treatment on autolysosome tubulation, cells were starved for 6 hr. Images were acquired using an Olympus FV-1000 confocal microscope.

### Autolysosome/Lysosome Purification and Labeling

Autolysosome/lysosome purification was performed with a lysosome isolation kit (LYSISO1, Sigma-Aldrich). The crude autolysosomes/lysosomes were obtained by differential centrifugation. And then the lysosome/autolysosome fraction was further purified from above preparation using optiprep gradient centrifugation. The fraction was collected and resuspended in extraction buffer containing protease inhibitor cocktail. To obtain pure lysosome/autolysosomes, this above fraction, containing autolysosomes/lysosomes, was diluted with 19% Optiprep density gradient medium solution, and overlaid with 16%, 12%, 8%, 5% and 3%. Optiprep solutions. After centrifugation, the fractions were analyzed by western blotting and TEM. The purified autolysosomes/lysosomes were collected and used for the *in vitro* assays.

The purified lysosomes/autolysosomes were washed twice by PBS. The pellet was suspended by 900 μl of 10 mM NHS-biotin solution and incubated for 30 min. The reaction was stopped by addition of 0.5 M TBS. The mixture were spinned at 20,000 × g at 4 °C, and resuspended with 100 μl of motility assay buffer for AFM assay. The pulldown assay was done by using streptavidin-conjugated beads, and the labeling efficiency was detected by western blotting.

### Liposome Preparation

Liposomes were prepared by dissolving a lipid mixture consisting of phosphatidylcholine (PC), phosphatidylethanolamine (PE) and Biotin-PE in chloroform followed by evaporation under a constant nitrogen stream. The lipids were rehydrated, and extruded through a 100 nm or 1000 nm pore polycarbonate filter using mini-extruders from Avanti Polar Lipids.

### Imaging

For live-cell imaging, transfected cells were re-plated in Lab-Tek chambered cover-glass before imaging, and cells were maintained at 37 °C with 5% CO_2_. Images were acquired using an Olympus FV-1000 confocal microscope. For lysosome movement assay in KIF5B KO cells, NRK cells with KIF5B KO were cultured in ø 3.5 cm cover-glass before imaging. 1 μM LysoTracker®Red DND-99 was added into culture medium and then the cells were maintained for 20 min at 37 °C with 5% CO_2_. Images were taken by using UltraView Vox Spinning Disk Confocal on Olympus IX83 Microscope.

### Protein Expression, Purification and Labeling

The dimeric kinesin construct (K_560_), with a C-terminal His tag and an Avi tag for biotinylation, was expressed in *E. coli* BL21 cells. Protein expression was induced with 0.1 mM IPTG at cell densities between OD600 ≈ 0.5–1.0, and the cells were harvested after 14 to 18 hr. Cells was lysed and protein was purified on Ni-NTA agarose column. To biotinylate the Avi-tagged K_560_, a mixture containing 38 μM Avi-tagged K_560_, 10 mM ATP, 40 μM d-Biotin and 25 μg/ml BirA was incubated in a 30 °C water bath for 30 min. Pulldown assays were conducted on biotinylated K_560_ (K_560_-biotin) using streptavidin beads, and the labeling efficiency was determined by SDS-PAGE.

For full-length KIF5B, proteins were expressed by using sf9 cells. Sf9 cells were grown to a density of ~2 × 10^6^ cells/ml, and incubated with virus containing full-length KIF5B construct. After transfecting 60 hr, cells were lysed by two freeze-thaw cycles. The soluble fraction was collected at 44,000 × g for 40 min, and the fraction was added to Ni-NTA agarose column. The resin was washed by 20 mM Tris-HCl, 0.5 M NaCl, 20 mM imidazole, pH 8, followed by 20 mM Tris-HCl, 1 M NaCl, pH 8. His_6_-tagged KIF5B was eluted with 20 mM Tris, 0.1 M NaCl, 250 mM imidazole, pH 8. Finally, the eluate was concentrated and stored in 50 μM HEPES-KOH, pH 7.4, 300 mM NaCl, 1 mM MgCl_2_, 10% (w/v) sucrose, 50 μM ATP.

### *In Vitro* Reconstitution of Autolysosome/Lysosome Tubulation

The tubulation assay was performed by using flow chambers as described before^6^. The channels were incubated with 10 μg/ml anti-tubulin antibody to allow microtubules to be immobilized on the coverslips, followed by 3 mg/ml casein to block the surface. For K_560_-biotin-induced liposome tubulation, 0~80 nM K_560_-biotin was incubated with 0.125 mg/ml streptavidin and 28 μg/ml biotin-liposomes. For autolysosome/lysosome tubulation, 80 nM full-length KIF5B was incubated with ~0.3 mg/ml autolysosomes/lysosomes. The motor-coated vesicles were introduced into the microtubule-coated flow chambers, and then 60 μl of ATP solution containing 20 μM ATP, 20 μM taxol, 1 mg/ml casein, an ATP regeneration system and an oxygen scavenger system were added to the chambers. The formation of tubes was visualized by using a Nikon TIRF microscope.

### FEISEM

For field emission in-lens scanning electron microscopy (FEISEM), autolysosomes were incubated on glass chips at 37 °C for 1 hr, and then fixed with 2% glutaraldehyde in PBS buffer at room temperature for 30 min. The samples were then rinsed and post-fixed with 1.0% OsO_4_ in 0.1 M sodium cacodylate buffer at room temperature for 20 min. After dehydration in a graded ethanol series (30%, 50%, 70%, 90%, 100%, 100%, 10 min each), the samples were transferred to Arklone for critical-point drying using highest-purity CO_2_ in a Hitachi HCP-2 Critical Point Dryer. The samples were then coated with 4 nm gold in a Hitachi E-1045 ion sputter coater and viewed in a Hitachi scanning electron microscope S4800 at a 6 kV accelerating voltage.

### AFM Assay

A home-made atomic force microscopy (AFM) built and calibrated as previously described^20^ was used to measure the tubulation of vesicles. The cantilever tips (Bruker AFM probes, MLCT) and the polystyrene Petri dish surface were incubated with 0.5 mg/mL streptavidin (Sigma Aldrich, S4762) at 4 °C overnight. After rinsing with BRB80 buffer (pH 6.8), the cantilever tips were incubated for 15 min at room temperature in BRB80 buffer with 0.1% BSA to block nonspecific adhesion. To capture vesicles, the streptavidin–precoated Petri dish was rinsed three times with BRB80 buffer and incubated with four different biotinylated vesicles (100 nm- and 1 μm-liposome, lysosome and autolysosome) for 30 min at room temperature, respectively. The Petri dish was again rinsed three times with BRB80 buffer and filled with 5 mL BRB80 buffer with 0.1% BSA.

The AFM force-ramp experiments were performed by repeatedly bringing the Petri dish into contact with the cantilever tip for 0.5 s to allow streptavidin on the tip to capture biotinylated vesicle, then retracting at a speed of 200 nm/s to pull for the tubulation of vesicle. The signal of the tip deflection was recorded to calculate the forces during tubulation. According to the previously reported results^15^,^16^, the value of the first peak force (initiating force) in each of force-time curves was collected for statistical analysis. The histograms of the initiating forces were fitted with Gauss distribution. The maximum force from the fitting was taken as the most probable initiating force.

## Additional Information

**How to cite this article**: Su, Q. P. *et al*. Vesicle Size Regulates Nanotube Formation in the Cell. *Sci. Rep*. **6**, 24002; doi: 10.1038/srep24002 (2016).

## Supplementary Material

Supplementary Information
