# Corrigendum: Vesicle Size Regulates Nanotube Formation in the Cell

**DOI:** 10.1038/srep40108

**Published:** 2017-02-09

**Authors:** Qian Peter Su, Wanqing Du, Qinghua Ji, Boxin Xue, Dong Jiang, Yueyao Zhu, He Ren, Chuanmao Zhang, Jizhong Lou, Li Yu, Yujie Sun

Scientific Reports 6: Article number: 2400210.1038/srep24002; published online: 04 07 2016; updated: 02 09 2017

He Ren and Chuanmao Zhang were omitted from the author list in the original version of this Article. This has been corrected in the PDF and HTML versions of the Article, as well as the Supplementary Information that now accompanies the Article.

The Author Contributions section now reads:

Q.P.S., W.D., Y.S., L.Y. and J.L. designed the research; Q.P.S., W.D., Q.J., Y.Z. and D.J. performed the research experiments; H.R. and C.Z. performed the FEISEM experiments. Q.P.S., W.D. and B.X. analyzed the data; Q.P.S., Y.S., L.Y. and J.L. wrote the manuscript.

