# Supplementary material for: Vesicle Size Regulates Nanotube Formation in the Cell
Source: Sci Rep. 2016 Apr 7;6:24002. doi: 10.1038/srep24002 (PMC4823757; doi:10.1038/srep24002)
Supplement: Supplementary Information [file srep24002-s1.docx]

**Supplementary Materials**

**Vesicle Size Regulates Nanotube Formation in the Cell**

**Qian Peter Su^1^, Wanqing Du^2^, Qinghua Ji^3,4^, Boxin Xue^1^, Dong Jiang^2^, Yueyao Zhu^2,6^, He Ren^5^, Chuanmao Zhang^5^, Jizhong Lou^3,4,*^, Li Yu^2,*^ and Yujie Sun^1,*^**

1 State Key Laboratory of Membrane Biology, Biodynamic Optical Imaging Center, School of Life Sciences, Peking University, Beijing 100871, China

2 State Key Laboratory of Membrane Biology, Tsinghua-Peking University Joint Center for Life Sciences, School of Life Sciences, Tsinghua University, Beijing 100084, China

3 Laboratory of RNA Biology, Institute of Biophysics, Chinese Academy of Sciences, Beijing 100101, China.

4 University of Chinese Academy of Sciences, Beijing 100049, China.

5 School of Life Sciences, Peking University, Beijing 100871, China.

6 Present Address: Department of Biology, University of Pennsylvania, Philadelphia, PA 19104-6018, USA

* Correspondence: Yujie Sun: sun_yujie@pku.edu.cn

Li Yu: liyulab@mail.tsinghua.edu.cn

Jizhong Lou: jlou@ibp.ac.cn

**This PDF file includes:**

**Supplementary Text**

**Figs. S1 to S4**

**Estimation of the force for nanotube formation of small-scaled autolysosomes and lysosomes**

To be on the ball, we must realize that the surface tension $\sigma$ now is the function of tube length L for small-scaled vesicle, of which the diameter is in the range of 50 nm to 200 nm. For a straight forward understanding, we define $\sigma_{effect}$ to simplify analysis, ignoring the shape distortion from tube-on-ball. Analog to the works done by Imre Derenyi et al (Derenyi, I., F. Julicher, and J. Prost, Formation and interaction of membrane tubes. *Phys Rev Lett*, **88**, 238101-1-4 (2002).), for small vesicles the free energy of the tube system could be expressed as following:

$$F_{tube}=\left[ \frac{k_{c}}{2{r_{0}}^{2}}+\sigma_{effect} \right]2\pi r_{0}L-fL$$

where $r_{0}$represents the radius of tube and L represents the tube length. Taking

$$\frac{{\partial F}_{tube}}{\partial r_{0}}=0 \frac{{\partial F}_{tube}}{\partial L}=0$$

We get

$$\left( 2\tau_{0}{R_{0}}^{2}+k_{c} \right)r_{0}^{2}+2k_{s}Lr_{0}^{3}-k_{c}R_{0}^{2}=0$$

$$f=2\pi k_{c}/r_{0}$$

We can see that for the same tube length L, the smaller the vesicle is, the more force it needs to maintain the tubulation. For a small-scaled vesicle during the tubulation process, the force against the increase of surface tension is approximately scale with the tube length as $f\sim\sqrt[3]{L}$.

Next we calculate the surface tension for a given tube length (L = 100 nm) and make a simple estimation for other parameters. For an autolysosome vesicle, experimental results indicated that $R_{0}=40 nm$, $f_{0}=20 pN{,R_{v}=1 um, L}_{0}=$3 um, from which we get (Derenyi, I., F. Julicher, and J. Prost, Formation and interaction of membrane tubes. *Phys Rev Lett*, **88**, 238101-1-4 (2002).)

$$k_{c}=127.4 pN\cdot nm \sigma= 0.04 pN/nm$$

With the assumption that $\sigma=k_{A}\cdot\frac{\Delta a}{a_{0}}{, a}_{0}=4\pi{R_{v}}^{2}$, $\Delta a=2\pi R_{0}L_{0},$ we get

$k_{A}=$0.67$pN\cdot nm$

We assume that $k_{c} \mathrm{and}k_{A}$of a lysosome vesicle are the same with that of an autolysosome. Then

$$\Delta a=2\pi\sqrt{\frac{k_{c}}{2\sigma}}L_{0}， a_{0}=4\pi{R_{v}}^{2}， \sigma=k_{A}\cdot\frac{\Delta a}{a_{0}}$$

For a nanotube of L = 100 nm, we get

$$\sigma= \left( \frac{k_{A}^{2}k_{c}L_{0}^{2}}{8R_{v}^{4}} \right)^{\frac{1}{3}}= 0.089 pN/nm$$

and

$$R_{0}=\sqrt{\frac{k_{c}}{2\sigma}}=26.8 nm f_{0}=2\pi\sqrt{2\sigma k_{c}}=29.9 pN$$

The estimated force is close to that measured with AFM (**Fig. 4**).

**Discussion on the effects that cause the size-dependence of vesicle tubulation**

Indeed, the size-dependence of vesicle tubulation may be resulted from several effects, including the motor density, number of engaged motors, counter force, and the increased excess surface area due to tubulation, which are not mutually exclusive. As a single kinesin motor can apply up to 6 pN force (Leduc, C., et, al. Cooperative extraction of membrane nanotubes by molecular motors. *Proc. Natl. Acad. Sci. U. S. A.* **101**, 17096-17101 (2004).) while it takes about 20 pN to initiate a tubule from a lysosome or autolysosome (**Fig. 4g-j**), the tubulation process therefore requires several motors to work together. Although the *in vitro* tubulation assay indicates that a higher motor density was indeed able to increase the tubulation percentage for the 500-1000nm liposomes, the increased kinesin concentration did not change the tubulation probability for the 100-200nm small liposomes (**Fig. 3d**). Therefore, the size dependence is not due to the motor density.

Disregard the motor density, the size-dependence may also be due to the number of engaged motors as a geometrical consequence. Given the diameter of microtubule is 25 nm and the nanotubule size is ~80 nm (Leduc, C., et, al. Mechanism of membrane nanotube formation by molecular motors. *Biochimica et Biophysica Acta* **1798**, 1418-1426 (2010).) the area of the tubule bud-microtubule interface can be estimated as a spherical cap about 500 nm^2^, which may allow 5 kinesin motors to bind if we assume a kinesin occupies a 10x10 nm^2^. This number of engaged motors is enough to pull tubules out of both lysosome and autolysosomes. Thus, the number of engaged motor is not the deterministic factor for the size-dependence effect.

A counter force must be present on the vesicle when kinesin motors are pulling a tubule out of the vesicle. The counter force in vivo may be caused by the hydrodynamic friction and the cytoskeleton network (the cytoplasm is more like a gel with a mesh size of only ~50nm, Lubyphelps, K., et, al. Hindered diffusion of inert tracer particles in the cytoplasm of mouse 3T3 cells. *Proceedings of the National Academy of Sciences of the United States of America.* **84**, 4910-4913 (1987).) and may impose more dragging force on autolysosomes than lysosomes. Still, as our *in vitro* tubulation assay largely diminished the dragging effect caused by the mesh of the cytoplasm, it suggests that the dragging force would not be the sole reason that causes the size-dependence of vesicle tubulation.


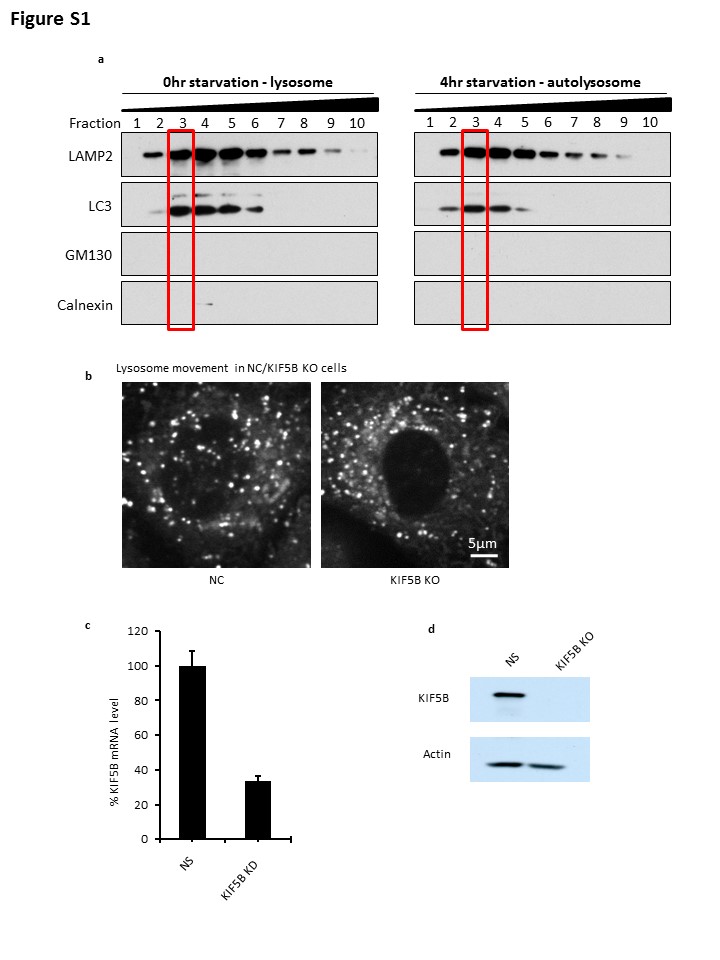


**Figure S1**

**(a)** NRK cells were straved for 0 h and 4 h, homogenized and then centrifuged in OptiPrep density gradient medium. Lysosme/autolysosome fractions was collected and subjected to the secondary OptiPrep density gradient medium. Fractions were analysized by western blotting with antibodies against LAMP2, LC3, GM130 and Calnexin. 1 is the top fraction. The outline indicated the pure LAMP2 and LC3-positive fraction. **(b)** Fluorescent images of lysosome movement in NS- and KIF5B-RNAi cells. **(c)** KIF5B mRNA level was analyzed by qPCR in NS- and KIF5B-RNAi cells. **(d)** KIF5B protein level was analyzed by western blot in NS- and KIF5B-RNAi cells.


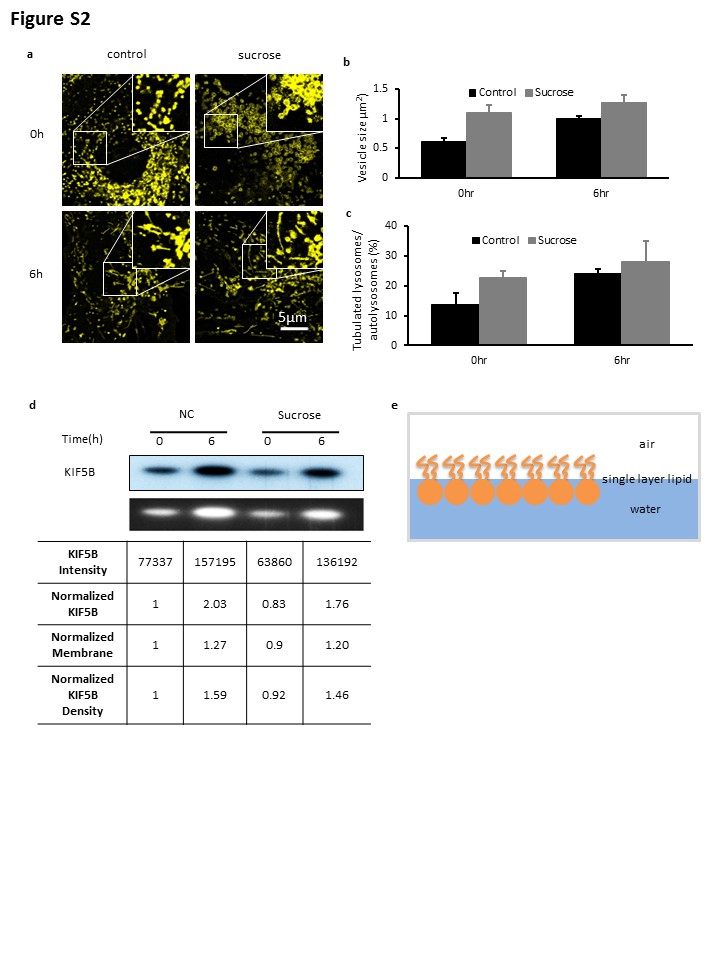


**Figure S2**

**(a)** Fluorescent images and zoom-in areas of Lamp1-positive vesicles in control and sucrose swelling cells after 0hr or 6hr starvation. **(b)** Vesicle sizes in (a). **(c)** Tubulation ratio of lysosomes/autolysosomes in (a) **(d)** Western blot towards kif5b in cells from (a) and its quantification. **(e)** Schematic diagram of single layer lipid measurement.


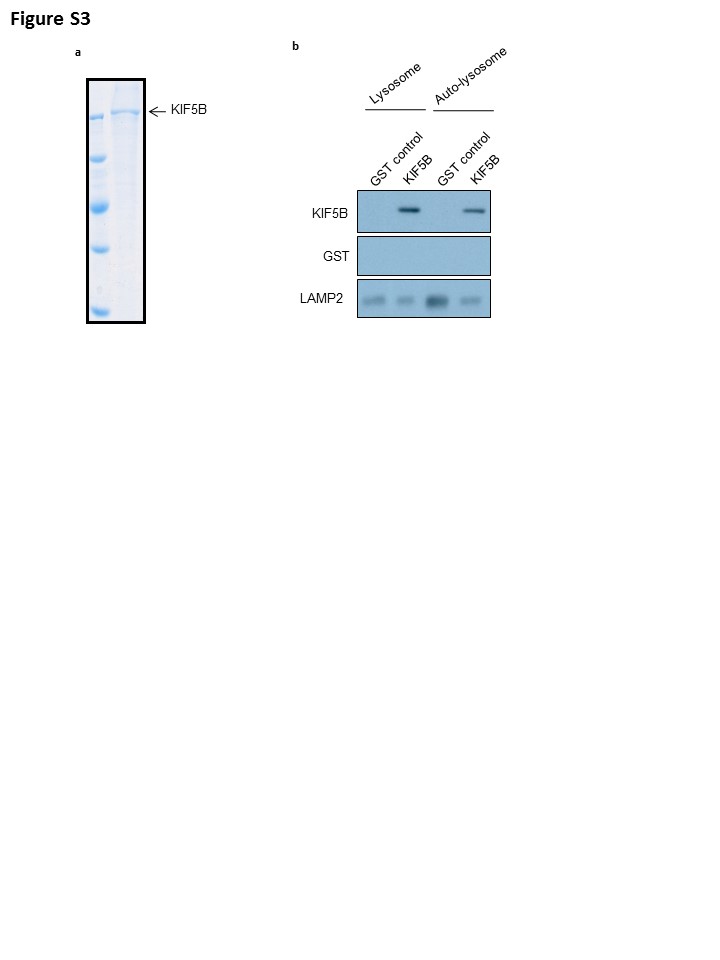


**Figure S3**

**(a)** Full-length KIF5B was expressed using the Bac-to-Bac expression system, and the purity of KIF5B was analyzed by Coomassie staining. **(b)** Purified lysosomes and autolysosomes were incubated with full-length KIF5B, washed, and analyzed by western blotting with antibodies against His tag, GST and LAMP2.


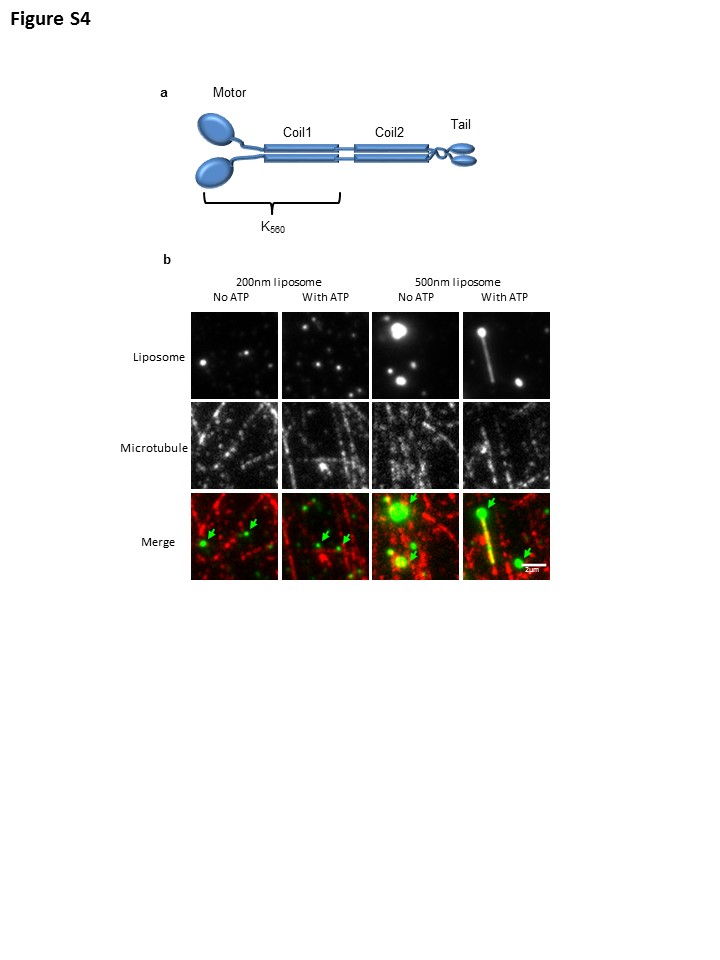


**Figure S4**

**(a)** Schematic diagram of the KIF5B protein, showing the motor domain (K_560_) used in this study. **(b)** two-color fluorescence images show that the tubules are pulled along the microtubule tracks.
